# Utilization of a Parental Approach to Informed Consent in Intravenous Tissue Plasminogen Activator Administration Decision-Making: Patient Preference and Ethical Considerations

**DOI:** 10.1155/2019/9240603

**Published:** 2019-09-05

**Authors:** Ann M. Murray, Ashley B. Petrone, Amelia K. Adcock

**Affiliations:** ^1^Department of Neurology, West Virginia University, Morgantown, WV 26506, USA; ^2^Department of Pathology, Anatomy and Laboratory Medicine, West Virginia University, Morgantown, WV 26506, USA

## Abstract

**Objective:**

While administration of intravenous tissue plasminogen activator (IV-tPA) is the standard of care in acute ischemic stroke and has been shown to have statistically significant benefit, there can also be potentially life-threatening complications; however, there is no standard informed consent approach. The purpose of this study was to present a parental, technical, and general model of informed consent for IV-TPA and to determine which approach was preferred.

**Methods:**

Survey respondents were asked to hypothetically decide whether or not to provide consent for their family member to receive IV-tPA. Respondents were presented with 3 informed consent models: one emphasizing parental qualities, one emphasizing statistical data, and one representing a general consent statement. After being presented each model, the respondents had to select their preferred consent model, as well as rate their level of agreeability toward their family member receiving the medication following each approach.

**Results:**

The results of 184 surveys showed respondents were equally as likely to give consent for their family member to receive IV-TPA following all three approaches; however, respondents were significantly more likely to prefer the parental approach compared to a technical or general approach.

**Conclusion:**

Our results indicate that while paternalism is generally discouraged in the medical community, some degree of parental language may be preferred by patients in tough decision-making situations toward consent to receive medical interventions.

## 1. Introduction

The term “Informed consent” is credited to attorney Paul G. Gebhard, as part of a medical malpractice case in the United Stated in 1957 [1]. However, the idea that physicians should fully inform patients about medical details in order to aid in their care dates back as far as 1849 *to* the American physician Worthington Hooker [1]. This concept was neither popular nor adopted into practice at that time [1]. In fact, in 1847, the American Medical Association published the *American Medical Association Code of Medical Ethics* which supported the idea that patients had a right to the truth except when physicians felt like they could improve care by withholding information [1]. This paternalistic, now also termed parental approach, was the standard practice model for medical decision-making until the concept of shared decision-making was introduced and first mentioned in the literature in 1982 [2]. Since then, there have been numerous publications supporting the importance of shared decision-making in both patient satisfaction and outcomes, and the shared decision-making model is now the standard of care for all decision-making in medicine [3]. It is important to differentiate shared decision-making *from* the formal informed consent used in research settings since the Nuremburg Code was established in 1947 [4]. There is a lot of overlap between informed consent and shared decision-making, but the difference lies in the extent the patient is actively involved in each process. Informed consent is the legal standard obligation of a healthcare provider to educate patients of the potential benefits and harms of any procedure or treatment, while shared decision-making highlights the importance of a thorough discussion of all options available to a patient allowing the patient to direct the treatment decision that is most consistent with his or her values. Despite widespread acceptance of shared decision-making and its adoption into practice, very limited data are available on applying these models in an emergency setting.

While administration of intravenous tissue plasminogen activator (IV-tPA) is the standard of care in acute ischemic stroke and has been shown to have statistically significant benefit with minimal risk in eligible patients, there can also be potentially life-threatening complications [5]. As such, many clinicians seek verbal informed consent prior to its administration; however, there is no standard IV-tPA consenting protocol. Often, clinicians adopt a statistical approach for IV-TPA informed consent by presenting risk/benefit statistics reported in the National Institute of Neurological Disorders and Stroke tPA Stroke Study; however, there are currently no data available to help understand if this approach aids in patient knowledge or confidence in decision-making, from the patients' perspective, in the setting of consenting for IV-tPA.

The purpose of this study was to present three distinct informative paragraphs/approaches that may be used in IV-tPA informed consent, and each paragraph used language and content of either a parental, technical, or general model of informed consent. After presented with all three models, respondents were asked to indicate which model was easiest to understand or would have the largest impact on IV-tPA decision-making for themselves or a family member. Our hypothesis is that the majority of respondents would prefer a parental approach, compared to a technical or general approach.

## 2. Methods

This study was approved by the West Virginia University Institutional Review Board. A voluntary survey was distributed to West Virginia University Hospital cafeteria adult visitors, comprising hospital employees, healthy patients, and visitors, in a hospital setting. The survey prompted respondents to imagine that their family member was experiencing an acute ischemic stroke, and they must decide whether or not to consent for their family member to receive IV-tPA. Respondents were presented with three distinct informative paragraphs/approaches that may be used in IV-tPA informed consent, and each paragraph used language and content of either a parental, technical, and general approach to informed consent. The survey text for each approach is provided in Table 1.

Respondents were asked to indicate a single, preferred informed consent approach, as well as rate their level of agreeability toward their family member receiving the medication on a 5-increment scale (definitely agreeable, probably agreeable, undecided, probably not agreeable, definitely not agreeable) for each of the three informed consent approaches, regardless of preference. Further, responses of “definitely agreeable” and “probably agreeable” were considered overall agreeable, a response of “undecided” considered uncertain, and responses of “definitely not agreeable” and “probably not agreeable” considered overall not agreeable.

### 2.1. Statistical Analysis

Descriptive statistics were used to compare the respondent demographics—gender and education level. Survey responses were recorded as frequencies, and a chi-square analysis was used to detect differences in the proportions of approach preference and overall agreeableness toward each approach.

## 3. Results

A total of 184 respondents completed the survey. Of the respondents, 62 were male (34%) and 122 were female (66%). Respondents were also asked to indicate his or her highest education level—7 did not complete high school (4%), 57 completed high school only (31%), and 120 received a bachelor's degree or some other form of education beyond high school (65%) (Table 2).

Respondents were equally as likely to give consent for their family member to receive IV-TPA following all three approaches, with 75 percent agreeable following a technical approach, 84 percent agreeable following a parental approach, and 85 percent agreeable following a general approach (Table 3). Further, overall agreeability toward any approach did not differ on the basis of gender nor education level.

Although there were no differences in overall agreeability between any of the three scenarios, respondents were significantly more likely to prefer the parental approach compared to a statistical or general approach (*p* < 0.001), as 55 percent of respondents indicated parental approach as their preferred scenario (Table 3). Not only did a significant majority of respondents indicate that a parental approach was their preferred approach, this approach was associated with the lowest proportion of uncertainty. Only four percent of respondents responded “undecided” regarding tPA administration following the parental approach, whereas 14 percent of respondents responded “undecided” following both the statistical and general approaches (Table 3). Lastly, neither scenario preference nor uncertainty differed on the basis of gender nor education level.

## 4. Discussion

The overall purpose of our study was to determine if a parental approach to informed consent was the preferred approach to informed consent, associated with the highest degree of confidence in IV-tPA administration decision-making, compared to a statistical or general approach. Although respondents were equally as likely to give consent for their family member to receive IV-TPA following all three approaches, respondents were significantly more likely to prefer the parental approach compared to a statistical or general approach. Furthermore, a parental approach was associated with the lowest proportion of uncertainty, indicating the highest degree of confidence in decision-making compared with the statistical or general approach.

Modern medical education tends to discourage physician paternalism while emphasizing the importance of patient autonomy and beneficence; however, these two principles are often at odds with each other. How can a patient or their surrogate have total autonomy and possess the requisite medical knowledge to make a decision that truly promotes beneficence above all else, especially in a time-sensitive case such as acute ischemic stroke? Perhaps some form of paternalism may be necessary to resolve this paradox, and our results demonstrate the influence that parental language can have in obtaining informed consent for IV-tPA. It is crucial to develop a more standardized approach or language utilized in consent for IV-tPA, with consideration for the ethical and legal aspects of medical-decision-making, to ensure that patients are provided the statistical risk information, while using some parental language to allow patients or their caregivers to make the most unbiased, informed decision.

The principle of shared decision-making tries to help bridge this divide between beneficence and autonomy by challenging physicians to provide medical details and treatment options. This encourages patients to make medical treatment decision themselves, without personal bias from the provider. Healthcare providers are coached to make recommendations to patients based on medical knowledge but are often discouraged to incorporate personal opinions on which decision they feel a patient should make. Fundamentally, shared decision-making requires the patient to be the center of the decision-making process and places emphasis on the patient's personal values and beliefs [6]. Once personal opinion from the provider is implemented in the decision-making process, the model shifts from shared decision-making toward a more parental approach, which is heavily influenced by the provider's own personal values and beliefs which could differ from the patient. In a comparison study done by Murray et al. among different models of decision-making, 62% of respondents preferred shared decision-making while only 9% preferred paternalism [7]. Like this study, most decision-making studies are done in a primary care setting leaving limited understanding of this process in an acute care setting. The results in our study are in stark contrast with most historically conducted decision-making model studies and emphasize the need for further research in this setting.

Although there are clearly preferences to style when it comes to decision-making in different scenarios, there still remains variability between preferences regardless of the setting.

Limitations to our study include the relatively small number of participants, single-site demographics, and recruitment on a healthcare campus. All of these factors may reduce the generalizability of our results, and future studies are warranted to support the generalizability of these results to other demographics. In addition, although we made every attempt to create scenarios that were purely “parental,” “statistical,” or “general,” the concurrent requirement to create a consenting scenario consistent with the one used in the real world meant that some elements of each are likely present. Further, the parental scenario also included language that was not only the opinion of the provider but also specific to our institution's perceived successful with tPA treatment.

Second, none of our scenarios explicitly addressed any specific adverse events associated with tPA administration, such as intracranial hemorrhage. Because no explicit discussion was part of the three scenarios in this study, it is impossible to predict how any specific risk would impact informed consent. For example, the question remains to be asked, would patients be more agreeable to a parental approach with or without an explicit discussion of risk, or more importantly, if this event would occur, would patients feel that their consent was truly informed? Both of these questions should be addressed in future studies.

Lastly, our hypothetical situation was, by design, a time-dependent high stakes medical decision. It is more likely that in such a situation, the patient or their surrogate will rely more heavily on the provider's advice, as compared to a time-independent situation where patients may receive a variety of treatment options and consider the information over a longer period. Therefore, it is difficult to generalize our findings to medical decision-making as it is applied to nonacute settings.

## 5. Conclusion

Despite the limitations of this study, our study represents a step toward identifying an optimal approach in informed consent, not just for IV-TPA administration, but that can be utilized in numerous acute decision-making scenarios in a clinical setting. Our results indicate that while paternalism is generally discouraged, some degree of parental language may improve patient agreeableness toward consent to receive medical interventions. While parental language seems to increase agreeableness, it is important to ensure that agreeability is on the basis of a thorough understanding of risk/benefit. It is our opinion that a combination of the parental and statistical approach would be the most ethical, informative approach; however, future studies are warranted to determine the optimal approach.

## Figures and Tables

**Table 1:** Survey text.

Scenario 1—General approach: “IV-TPA is an FDA-approved clot busting medication. Overall, your mother is more likely to benefit from this medication than be harmed from this medication. We would recommend you allowing us to give this medication to your mother”
Scenario 2—Technical approach: “IV-TPA is an FDA approved clot busting medication. Your mom has a 33% chance of improving from this medication and a 6% chance of harming her with this medication. If you took 100 people and gave them this medication 33 of them would get better, 60 of them wouldn't get better, but they wouldn't get worse, 6 of them would get worse but only minorly so, and 1 of them would potentially have a life-threatening complication. Overall, we feel as though your mom is more likely to benefit from this medication than be harmed, and we would recommend you allowing us to give your mom this medication”
Scenario 3—Parental approach: “IV-TPA is an FDA approved clot busting medication. Our team has given this medication to thousands of people over the years and our institution does an amazing job at keeping our patients safe when we give this medication, minimizing any potential harm it could cause. This is a clot busting medication, so the major risk is bleeding, but we feel as though the potential benefit significantly outweighs the minimal risk. I would recommend you letting us give this medication to your mother. I know this is an extremely hard position to be in, but if I was in your shoes, I would want to give this medication to my loved one”

**Table 2:** Respondent demographics.

Demographic	*N* (%)
Gender	
Male	62 (34)
Female	122 (66)
Highest education	
Did not complete HS	7 (4)
Completed HS	57 (31)
Some college	70 (38)
Bachelor's degree	50 (27)

**Table 3:** Scenario preference, agreeability, and uncertainty.

*N* (%)	Scenario 1—general	Scenario 2—technical	Scenario 3—parental
Preference	29 (16%)	53 (29%)	102 (55%)
Overall agreeability	156 (85%)	138 (75%)	154 (84%)
Uncertainty	25 (14%)	26 (14%)	7 (4%)

## Data Availability

The data collected in this study can be made available upon request.

## References

[1] Capron A. M. (1987). (Almost) everything you ever wanted to know about informed consent. [Review of: faden, RR and Beauchamp, TL. A history and theory of informed concsent. New York and Oxford: Oxford University press, 1986]. *Medical Humanities Review*.

[2] United States Office of the Federal Register (1983). Protection of human subjects; reports of the president’s commission for the study of ethical problems in medicine and biomedical and behavioral research-office of the assistant secretary for health, HHS. Notice of availability of reports. *Federal Register*.

[3] Stacey D., Bennett C. L., Barry M. J. (2011). Decision aids for people facing health treatment or screening decisions. *Cochrane Database of Systematic Reviews*.

[4] Ghooi R. B. (2011). The Nuremberg code-A critique. *Perspectives in Clinical Research*.

[5] National Institute of Neurological D, Stroke rt PASSG (1995). Tissue plasminogen activator for acute ischemic stroke. *The New England Journal of Medicine*.

[6] Murray E., Charles C., Gafni A. (2006). Shared decision-making in primary care: tailoring the Charles et al. model to fit the context of general practice. *Patient Education and Counseling*.

[7] Murray E., Pollack L., White M., Lo B. (2007). Clinical decision-making: patients’ preferences and experiences. *Patient Education and Counseling*.

